# The Case for Malnutrition Quality Measures and Nutrition-Focused Quality Improvement Programs (QIPs) in US Skilled Nursing Facilities

**DOI:** 10.3390/healthcare10030549

**Published:** 2022-03-16

**Authors:** Mary Beth Arensberg, Cory Brunton, Brenda Richardson, Scott Bolhack

**Affiliations:** 1 Abbott Nutrition Division of Abbott, Columbus, OH 43219, USA; cory.brunton@abbott.com; 2 Brenda Richardson LLC, Salem, IN 47167, USA; brenda@brendarichardson.com; 3 The Wound Care Center at Northwest Hospital, Tucson, AZ 85741, USA; sbolhack@arizona.edu

**Keywords:** skilled nursing facility (SNF), nutrition, malnutrition, quality improvement programs (QIPs), quality measures

## Abstract

As skilled nursing in the United States moves to a value-based model, malnutrition care remains a critical gap area that is associated with multiple poor health outcomes, including hospital readmissions and declines in functional status, psychosocial well-being, and quality of life. Malnutrition is often undiagnosed/untreated, even though it impacts up to half of skilled nursing facility (SNF) residents, and COVID-19 infections/related symptoms have likely further increased this risk. In acute care, malnutrition quality measures have been both developed/tested, and nutrition-focused quality improvement programs (QIPs) have been shown to reduce costs and effectively improve care processes and patient outcomes. Less is known about such quality initiatives in SNF care. This perspective paper reviewed malnutrition-related quality measures and nutrition-focused QIPs in SNFs and nursing home care. It identified that although the Centers for Medicare & Medicaid Services (CMS) has had a nursing home Quality Assurance and Performance Improvement (QAPI) program for 10 years and has had SNF quality measures for nearly 20 years, there are no malnutrition-specific quality measures for SNFs and very few published nutrition-focused QIPs in SNFs. This represents an important care gap that should be addressed to improve resident health outcomes as SNFs more fully move to a value-based care model.

## 1. Introduction

Skilled nursing facilities (SNFs) continue to account for the majority of spending for post-acute care services in the United States (US) [1]. Population growth of the US older adult segment (aged 65+) is projected to drive an estimated 50% increase in the number of older Americans needing skilled nursing care in the next decade, rising to about 1.9 million in 2030, up from 1.2 million in 2017 [2]. This growth comes at a time of significant change in coverage/reimbursement models for SNFs. The change is being led by government payors, particularly through the federal government’s Medicare program, which accounts for over 21% of spending on US long-term services and supports [3].

In skilled nursing care—as in all other areas of US healthcare—federal Medicare continues its progress toward value-based payment and away from traditional fee-for-service models. A significant shift occurred in October 2019, when the US Centers for Medicare & Medicaid Services (CMS) moved from the Resource Utilization Groups, Version IV (RUG-IV), to the Patient Driven Payment Model (PDPM). PDPM focuses on quality of care and patient outcomes, as opposed to quantity of provided services. Specifically, PDPM assigns each resident a case-mix classification based on five different components addressing acuity of illness and management needs, which then drives the daily reimbursement rate for that individual. CMS has commented that while the RUG-IV system reduced resident care to a single, typically volume-driven, case-mix group, PDPM focuses on the individual needs/goals of each resident [4].

One critical area of individualized resident need is nutrition, and the Federal Nursing Home Reform Act legally requires that nursing home meals meet the daily nutrition and special dietary needs of each resident [5]. Speech Language Pathology (SLP) and Non-therapy Ancillary (NTA) are the two PDPM case-mix adjustment components that include nutrition-related conditions (Table A1). Malnutrition is one of the diagnoses identified under NTA, but it is frequently undiagnosed in skilled nursing facilities, even as malnutrition impacts an estimated 35% to 50% of older residents in long-term care facilities [6], and COVID-19 infection and its related symptoms have likely increased malnutrition risk even further [7].

In addition to impacting individual resident needs, nutrition can also impact SNF business models. Tavakoli et al. [8] suggest malnutrition prevalence may have a direct impact on the risk adjustment of a long-term care facility’s outcome indicators, use of healthcare resources, and financial outcomes. They advocate that accurate prevention and diagnosis of malnutrition is important for effective resident outcomes and efficient institution resource utilization.

SNFs may be challenged by an aging population, as more people need supportive care but may have poorer health outcomes because of unrecognized, undiagnosed, and untreated malnutrition. Quality measures and quality improvement programs (QIPs) can help address these challenges and improve care processes to benefit both residents and SNFs. In acute care, malnutrition quality measures have been developed/tested and nutrition QIPs have been shown to be effective in identifying and intervening for malnutrition [9]. However, little is known about or has been reported on malnutrition quality measures, and nutrition-focused QIPs in skilled nursing care.

This perspective paper reviews malnutrition-related quality measures and nutrition-focused QIPs in SNFs and nursing home care. To provide context, it initially summarizes the problem of malnutrition in SNFs and outlines the CMS quality framework for nursing home care. It then summarizes our review and findings of SNF quality measures and nutrition-focused QIPs. The paper characterizes the gap in skilled nursing care at the intersection of value-based healthcare models and resident malnutrition and makes the case for how quality measures and nutrition-focused QIPs can help address this gap to benefit resident and SNF outcomes. 

## 2. The Problem of Malnutrition in Nursing Home Care

Age-related changes—appetite loss, limited ability to chew/swallow, polypharmacy, and cognitive/functional declines—commonly affect diet and nutrition. The most common form of malnutrition in older adults is protein-calorie malnutrition, which is associated with multiple poor health outcomes including decreased immunity, increased infection rates, delayed wound healing, and decreased respiratory and cardiac function, as well as increased healthcare costs [10]. Malnutrition is also a significant comorbidity affecting survival and healthcare costs in CMS beneficiaries with chronic diseases such as diabetes mellitus [11]. In 2020, it became clear that older adults in nursing home care were particularly vulnerable to COVID-19 infection [12]. Disease-related malnutrition can put immunocompromised individuals at a higher risk of contracting COVID-19 infection, and conversely, coronavirus infection can markedly increase malnutrition risk. Importantly, for residents with COVID-19, nutrition-focused care may improve outcomes [13].

In addition to poorer health outcomes, malnutrition in nursing homes is associated with declines in functional status and psychosocial well-being and can negatively influence quality of life [14]. It is not surprising then that measurement and documentation of malnutrition can help account for differences in the risk for adverse events and the resulting financial implications for SNFs [15]. Malnutrition is also linked to increased hospital readmissions [16], which is significant for SNFs because one in four patients discharged to a SNF is readmitted to the hospital within 30 days [17] and two-thirds of readmissions may be preventable [18]. Further, readmission rate is an important CMS quality measure that is tied to SNF Medicare reimbursement [19].

Nutrition intervention has been associated with improved health status and related cost savings across healthcare systems, including residents in nursing homes [20,21,22,23]. Early nutrition intervention can reduce readmissions and decrease malnutrition complication rates and costs of care [10]. A systematic review by Hugo et al. [24] concluded “supplements and food-based nutrition interventions in the aged care setting are clinically effective, have a low cost of implementation, and may be cost-effective at improving clinical outcomes associated with malnutrition.” Specific nutrition interventions such as oral nutrition supplements (ONS) have also been shown to be cost-effective relative to dietary advice in care homes [21].

Ideally, malnutrition is identified in other institutional or community settings even before individuals enter skilled nursing care. Yet this often does not occur. An estimated 20–50% of patients admitted to the hospital are malnourished [25] but less than 9% have a malnutrition diagnosis at discharge [26]. The prevalence of malnutrition in the community in the US is not well known or diagnosed [27]. Unfortunately, there is a gap in potential prevalence and diagnosis of malnutrition in SNFs too. Malnutrition may impact up to half of skilled nursing facility residents [6]; however, a recent claims analysis documented that even though malnutrition diagnosis claim rates have increased in recent years, the percent of malnutrition diagnoses was still less than 12% of claims for a specific set of Medicare SNF claims [28]. 

Under the PDPM, every resident admitted to the SNF requires an appropriate nutrition assessment within 5 days of the admission to identify all SLP and NTA comorbidities. Data from the Minimum Data Set (MDS) gathered on admission can help inform the nutrition assessment, but when malnutrition is identified, it must be documented as a diagnosis. If a correct malnutrition diagnosis is not recorded within the 5-day timeframe, treatment delays resulting in poorer patient outcomes can occur, and resources to effectively address nutrition needs may be impacted. Thus, more needs to be done to better identify as well as document malnutrition diagnoses in SNFs, including leveraging the SNF quality framework. 

## 3. US Centers for Medicare & Medicaid Services (CMS) Quality Framework in Nursing Home Care

CMS has a long-established framework for improving the quality of nursing home care, including a national Nursing Home Quality Initiative (NHQI) launched nearly two decades ago. The NHQI was built on a set of validated, publicly reported quality measures aimed at helping consumers and their families make more informed decisions in selecting a nursing home. Another NHQI goal has been making quality information publicly available to proactively motivate providers to address/improve their quality of care [29].

The key features of SNF value-based payments under CMS are (1) evaluation according to performance on a hospital readmission quality measure; (2) scoring on both improvement and achievement; (3) confidential quarterly feedback report on performance quality; and (4) earned incentive payments based on performance [30]. Rates of reimbursement are adjusted annually based on economic measures of cost increases/rule adjustments, and quality assessment is used for reimbursement incentives [31]. 

A different part of CMS’ quality framework is the Quality Assurance and Performance Improvement (QAPI) management system, which was established in response to provisions under the 2010 Affordable Care Act (ACA). The ACA requires nursing care facilities to adopt systems to continuously identify and correct deficiencies and promote and sustain performance improvement. CMS’ approach for executing this through the nursing home QAPI program is based on five key elements of effective quality management and implementation science:

1. Design and Scope;
2. Governance and Leadership;
3. Feedback, Data Systems, and Monitoring;
4. Performance Improvement Projects (PIPs);
5. Systematic Analysis and Systematic Action [32].

CMS defines a PIP as a “concentrated effort on a particular problem in one area of the facility or facility wide; it involves gathering information systematically to clarify issues or problems and intervening for improvements” [32]. As malnutrition care remains a problem area for SNFs, the PIP model—specifically nutrition-focused QIPs—may help examine and improve resident care and facility outcomes. Quality improvement through QIPs is intended to yield more efficient use of healthcare resources and staff while preserving focus on high quality care. CMS’ QAPI management system for nursing home care aligns with QIPs. For example, similar to PIPs, QIPs are systematic, formal approaches to analyze practice performance and to take action to improve performance. Strategies and tools for nutrition-focused quality improvements are available, and the likelihood for health and cost benefits is high [9,21,33,34,35,36,37]. Further, it has been identified that “evidence-based organizational strategies have the potential to improve resident care to a far greater extent than clinical interventions implemented by a single healthcare professional treating individual residents” [38].

## 4. Review of Malnutrition Quality Improvement in Nursing Home Care

Our review of malnutrition quality improvement in SNFs had two components: SNF quality measurement, and published, nutrition-focused QIPs in SNFs.

### 4.1. SNF Quality Measurement Review and Findings

CMS annually adopts and removes measures from its SNF quality reporting program [39]. In reviewing 2021 published CMS measures [19], we found no malnutrition-specific quality measures in the SNF quality reporting program. We then considered whether existing SNF quality measures include conditions/factors that may be related to malnutrition and could be impacted by early identification of malnutrition and interventions to correct it. Table 1 summarizes our findings of the SNF short stay (≤100 days post hospitalization) and SNF long stay quality measures that we identified as potentially related to malnutrition (Table 1).

### 4.2. SNF Nutrition-Focused QIP Review and Findings

We next reviewed English language literature from the last 10 years (2011–2021) to identify malnutrition and nutrition-focused QIPs and quality effectiveness process initiatives in SNFs/nursing home care. Key search terms included the following: SNFs, long-term care, assisted living, senior care, nursing homes, and nutrition-focused quality improvement/effectiveness (Table 2) as well as malnutrition-specific quality measures in SNFs.

We found 6 publications on nutrition-focused QIPs in skilled nursing facilities. The majority was published abstracts and most concentrated on efficacy/effectiveness versus clinical practice changes/process improvements (Table 3).

## 5. Discussion

We identified very few published nutrition-focused QIPs in SNFs, and the majority of the studies did not focus on US SNF populations. Among our findings were that Elia et al. [21] undertook a prospective, incremental cost-effectiveness analysis in older nursing home residents (≥80 years) randomized to receive either ONS or dietary advice for 12 weeks; their results showed that use of ONS was cost-effective relative to dietary advice. Simmons et al. [45] sought to enhance mealtime feeding assistance and between-meal snack delivery for residents with dementia and associated risk of low oral intake and unintentional weight loss; QIP strategies resulted in sustained improvements in eating and snacking.

Because of the limited literature base specific to nutrition-focused QIPs in SNFs, lessons from nutrition-focused QIPs in other care settings may be relevant, as poor nutrition status predisposes residents to falls and fractures, impaired skin integrity (pressure ulcers/injuries), and limited mobility [10]. One example of an effective nutrition-focused QIP model that could be adapted for use in SNFs is the Malnutrition Quality Improvement Initiative (MQii). The MQii has demonstrated that implementing a nutrition-focused QIP can enhance identification and management of patients who are malnourished [49] and has created an evidence-based malnutrition improvement toolkit for implementing improved practices [50]. In addition, the MQii has developed/researched a set of malnutrition electronic clinical quality measures [51] including a global malnutrition composite score measure [52]. The toolkit and malnutrition quality measures are specific to hospitals, but the MQii also supports implementing the model in other care settings and with other patient populations [35].

Nutrition-focused QIP models have been proven effective when they include malnutrition risk screening at admission, prompt initiation of ONS for at-risk patients, and nutrition education and follow-up. Specifically, a nutrition-focused QIP was associated with an overall cost-savings of over $4.8 million in the hospital setting [43] and a cost-savings of $2.3 million in the home health setting [22] driven by reduced healthcare resource use (combination of hospitalizations, emergency department visits, and outpatient visits) over the study periods of 90 days. For patients enrolled in supportive home-health care following a hospitalization, those in a nutrition-focused QIP experienced lower 90-day readmission rates and savings in healthcare costs ($1500/patient) compared to standard care control groups [22]. When implemented in the outpatient clinic setting, patients participating in the QIP were associated with an 11.6% reduction in healthcare resource use and net savings of $485 per patient treated [53].

Drawing on such literature, nutrition-focused QIPs in SNFs could impact multiple components of the nutrition care process to improve timeliness, effectiveness, and outcomes. We suggest several potential QIP areas for process improvement in SNFs (Table 4).

However, adjustment for systematic differences in SNFs that could impact uptake and embedding of new practices may be needed. For example, because many SNF residents have other comorbidities related to malnutrition, such as disability [54] and obesity [55], the QIP may need to focus on timely malnutrition screening and intervention as well as regular rescreening/monitoring and applicable adjustments in nutrition care plans.

In addition, interventions that may be suitable in other care settings, such as patient nutrition education, may have less of a role and other considerations such as addressing medications that can impact appetite, and avoiding needlessly restrictive diets may become primary areas of focus. Indeed, it is the position of the Academy of Nutrition and Dietetics that in post-acute care settings, older adults who prioritize quality of life/personal choice receive the least restrictive diet appropriate to meet their nutrition needs [16]. In addition, despite recognized needs, implementing nutrition-focused QIPs in nursing homes can be challenging due to constraints on time and staffing [56].

## 6. Making the Case for Malnutrition-Specific Quality Measures and Nutrition-Focused QIPs in Nursing Home Care

Although CMS has had a nursing home QAPI program for 10 years and has had SNF quality measures for nearly 20 years, our review identified that there are no malnutrition-specific quality measures for SNFs and very few published nutrition-focused QIPs in SNFs. Yet a case can be made that both are needed to better identify and intervene for malnutrition and benefit health outcomes as SNFs more fully move to a value-based care model and policymakers, healthcare organizations, and interdisciplinary teams all have a role.

### 6.1. Why SNF Malnutrition-Specific Quality Measures

The Improving Medicare Post-Acute Care Transformation Act of 2014 (IMPACT Act) requires development of quality measures [16]. Screening, assessment, diagnosis, and intervention for malnutrition are notably missing from current CMS SNF quality measures, even though malnutrition is common among residents and associated with increased risk for infections, readmissions, falls, impaired wound healing, pressure injuries, physical limitations and disabilities, and even death [10]. CMS specifically developed SNF quality measures to help consumers and their families make more informed decisions and motivate providers to address and improve quality of care. Given that malnutrition may impact up to half of residents in SNFs [6], what can be more fundamental than measuring quality of malnutrition care?

### 6.2. Why SNF Nutrition-Focused QIPs

There are several reasons why SNF nutrition-focused QIPs are needed. First, when malnutrition and other nutrition-related conditions are identified early, they can be part of the PDPM case-mix adjustment components, which identify complexity of care and resource needs. In addition, malnutrition impacts multiple, existing quality measures, including readmission, which is a key performance measure for SNF value-based care reimbursement.

Second, effective malnutrition care is an interdisciplinary process in SNFs. The medical complexity of residents in post-acute settings such as SNFs is increasing; for example, the percent of residents receiving total parenteral nutrition (TPN) is greater in SNFs today [57]. However, unlike acute care where registered dietitian nutritionists (RDNs) are available full time to provide malnutrition care, RDN presence in SNFs is more limited. The RDN must communicate effectively with the medical director to assure that malnourished residents are accurately diagnosed and have the appropriate documentation in the medical record. Yet because most SNFs only have an RDN onsite for a specific number of hours per month, the RDN must rely on the interdisciplinary team to identify residents at malnutrition risk and to implement and report on the effectiveness of nutrition interventions.

Nearly a decade ago, Dyck and Schumacher identified nurse leaders as playing pivotal roles in evidence-based clinical interventions for unintentional weight loss among older adults in long-term care [38]. Today, nurse leadership remains critical but may be further complicated by the staffing challenges many SNFs face—challenges amplified during the COVID-19 pandemic [58]—that can contribute to interdisciplinary team turnover and available staff having limited skills/training to recognize malnutrition risk. With the proper training and education, nurse-led nutrition-focused QIPs have been shown to produce positive outcomes, such as reduced time to ONS-initiation, reduced length of stay, and reduced probability of readmission [59]. Thus, one of the most important aspects of a nutrition-focused QIP is targeting the improved identification of malnutrition risk and more effectively communicating about malnutrition interventions, which could yield beneficial clinical process changes.

Third, because the PDPM model requires resident conditions be fully documented within 5 days, optimization of the malnutrition care process has become more important than ever, which is where nutrition-focused QIPs play a critical role. QIPs can help identify specific areas where the malnutrition care process or team communications may be breaking down and then test models for improvement. In acute care, improvement models include nursing screening and intervening for malnutrition, under the supervision of an RDN or physician, and using well-established electronic health record-cued algorithms [44]. Such models could be adapted for SNFs. Another important consideration is how transition of care data can be leveraged. One specific goal of the IMPACT Act is improved access to information by post-acute providers, which could benefit the exchange of nutrition-related data [16].

### 6.3. Opportunities for Development and Engagement

The next steps for developing SNF malnutrition quality measures and nutrition-focused QIPs align with the ongoing progress in nursing home care reform. Nutrition status was mentioned in the 2022 SNF QRP final rule as a condition that CMS may consider in future measure development efforts [60]. More recently, the Biden Administration announced multiple reforms to improve the safety and quality of care in the nation’s nursing homes. One of these reforms is to “Provide technical assistance to nursing homes to help them improve”, and it is noted that “CMS currently contracts with Quality Improvement Organizations that help providers across the health care spectrum make meaningful quality of care improvements. CMS will ensure that improving nursing home care is a core mission for these organizations and will explore pathways to expand on-demand trainings and information sharing around best practices, while expanding individualized, evidence-based assistance related to issues exacerbated by the pandemic” [61]. Quality malnutrition care is certainly an excellent “best practice” to share, and as identified earlier in this perspective review, nutrition was impacted by the pandemic.

In addition, nursing homes are routinely surveyed to assure they are in compliance with federal requirements, and CMS’ Requirements of Participation help clarify the intent of federal Medicare regulations [62]. While administrative priorities and the COVID-19 pandemic have delayed the full release of CMS’ latest (Phase 3) Requirements of Participation for SNFs, quality improvement practices and implementation remain major focuses for Phase 3, including that SNFs have ongoing, comprehensive QAPI programs addressing the full scope of care and services provided by the facility. RDNs working in nursing home care have suggested a range of nutrition-focused ideas on how to engage in facility QAPI programs, such as those related to improving quality measures (particularly those related to unplanned significant weight loss and pressure ulcers) and resident satisfaction [63].

## 7. Summary and Implications for Resident Care, Research, and Public Policy

As the US population grows older, more people with age-associated diseases and disabilities will increase the demand for skilled nursing care. Further, older age is associated with greater likelihood of worsening nutrition status, due to the merging influences of advancing age, poor dietary intake, and acute/chronic disease. In acute care, malnutrition quality measures have been both developed/tested, and nutrition-focused QIPs have been shown to reduce costs and effectively improve care processes and patient outcomes. Our review identified that even though there has long been a SNF quality improvement framework, there still are no malnutrition-specific quality measures and very few published nutrition-focused QIPs in SNFs. Reasons for this are unknown and represent an important area for future research. More importantly, the gap in malnutrition quality care continues to impact the health, independence, and quality of life for older people. Thus, policymakers should include malnutrition-focused quality measures as part of SNF quality reporting programs, and care providers should routinely include malnutrition care in facility QAPI programs. Taking these steps can harness the benefits of improved nutrition status as a pathway for reducing readmissions, increasing quality of life, and better supporting SNF value-based care.

## Figures and Tables

**Table 1:** Skilled Nursing Facility (SNF) Centers for Medicare & Medicaid Services (CMS) quality measures [19] potentially related to malnutrition.

SNF Short Stay (<100 Days) Quality Measures	SNF Long Stay (>100 Days) Quality Measures	How Malnutrition Is Related
% short-stay residents who were re-hospitalized after a nursing home admission	# of hospitalizations per 1000 long-stay resident days	Associated with increased hospital readmission [40]
% short-stay residents who have had an outpatient emergency department (ED) visit	Outpatient ED visits per 1000 long-stay resident days	Associated with increased ED visits [41]
% SNF residents with pressure ulcers that are new or worsened (SNF QRP)	% long-stay high-risk residents with pressure ulcers	Associated with increased risk for pressure ulcers and difficulty in healing pressure ulcers [42]
Rate of successful return to home and community from a SNF (SNF QRP)	% long-stay residents whose ability to move independently worsened	Associated with increased frailty and disability [27]
% short-stay residents who improved in their ability to move around on their own	% long-stay residents whose need for help with daily activities has increased	Associated with increased frailty and disability [27]
% SNF residents who experience one or more falls with major injury during their SNF stay (SNF QRP)	% long-stay residents experiencing one or more falls with major injury	Associated with increased falls risk and increased frailty and disability [27]
Rate of potentially preventable hospital readmissions 30 days after discharge from a SNF (SNF QRP)		Associated with increased hospital readmission [43]
Medicare Spending Per Beneficiary (MSPB) for residents in SNFs (SNF QRP)		Associated with increased healthcare costs [22,43]
	% of long-stay residents who lose too much weight	% of weight loss is a diagnostic indicator of malnutrition [44]

% Percent; # Number; QRP: Quality Reporting Program.

**Table 2:** Key search terms for research review of malnutrition and nutrition-focused quality improvement programs (QIPs) and quality effectiveness process initiatives in skilled nursing facilities (SNFs).

String	Terms
Setting	Long-term care, skilled nursing facilities, assisted living, senior care, nursing homes
Nutrition	Nutrition, nutritional, meal, diet, dietary programs, meal or feeding regime, protein, menus, oral nutrition supplement (ONS), oral nutrition intervention, wound care intervention
Efficacy	Efficacy, effectiveness, efficient, efficiency
Quality/Intervention	Quality improvement, intervention, improvement, improving, reform, pilot programs

**Table 3:** Summary of publications identified through a research review of published findings in last 10 years on malnutrition/nutrition-focused quality improvement program (QIPs) and quality effectiveness process initiatives in skilled nursing facilities (SNFs).

Publication	Methodology	Conclusions
A quality improvement system to manage feeding assistance care in assisted-living [45]	Describes the feasibility of a quality improvement system to manage feeding assistance care processes in an assisted living facility Assessed feeding assistance care quality during and between meals for 12 consecutive months for 53 residents Direct care staff received feedback about the quality of assistance and consistency of between-meal snack delivery for residents with low meal intake and/or weight loss	The quality improvement system resulted in sustained levels of mealtime feeding assistance and between-meal snack delivery and a low prevalence of weight loss among assisted living facility residents
Effects of nutritional intervention in long-term care in Korea [46]	Measured the effects of implementing a nutrition intervention involving nursing staff education, facilitation, and a shared algorithm (NIEFA) for institutionalized older adults in Korea A pre-post design was used and included 2 long-term care facilities	Implementation of NIEFA in a long-term care facility is effective in improving the nutrition status of older adults with or at risk of malnutrition Significant improvements were found in the intervention group’s daily energy intake, total lymphocytes, hemoglobin levels, hematocrit levels, and Mini Nutritional Assessment scores
Cost-effectiveness of nutrition intervention in long-term care [23]	Research staff provided supplements or snacks consistent with each participant’s diet orders twice per day 5 weekdays per week, for 24 consecutive weeks Research staff provided both intervention groups with assistance according to a standardized protocol to enhance independence and intake	Oral liquid nutrition supplements and snack offers were efficacious and cost-effective in increasing caloric intake when coupled with assistance to promote consumption
Cost-effectiveness of oral nutritional supplements in older malnourished care home residents [21,47]	Examined whether oral nutrition supplements (ONS) are cost-effective relative to dietary advice 104 older care home residents (88 ± 8 years) without overt dementia were randomized to receive either ONS or dietary advice for 12 weeks	The study indicated that ONS can improve quality of life and nutrition intake more effectively than dietary advice alone Use of ONS in care homes are cost-effective relative to dietary advice
A hospital-based multidisciplinary approach improves nutritional status of the elderly living in long-term care facilities in middle Taiwan [48]	In the intervention group, a case management model, with a hospital-based, multidisciplinary care-team, including a medical doctor, nurse, dietitian, and pharmacist, was adopted for each participant A dietitian gave each resident their dietary suggestions, with follow-up every 2 weeks	The nutrition status of elderly residents living in long-term care facilities in Taiwan improved effectively with a hospital-based multidisciplinary approach

**Table 4:** Potential areas and goals for nutrition-focused quality improvement programs (QIPs) to target malnutrition care process improvement in skilled nursing facilities (SNFs).

Potential QIP Area for Process Improvement	Potential QIP Goal for Process Improvement
Nutrition screening, assessment, diagnosis	Nutrition screening within 24 h of resident admission Nutrition assessment (of residents identified at malnutrition risk) and corresponding malnutrition diagnosis, within 4 days of positive malnutrition screen Access to upstream resident records that may identify malnutrition-related conditions and risk factors
Documentation of malnutrition diagnoses	Accurate documentation and coding of appropriate malnutrition diagnoses, within 5 days of resident admission
Communication of nutrition status	Results of nutrition screenings, assessments, and diagnoses communicated to interdisciplinary care team within 24 h of findings Results of all nutrition screenings, assessments, and diagnoses included as part of medical record accessible by all members of care team and residents’ family members, including at discharge
Documentation of nutrition care plan and nutrition interventions	Documentation of nutrition care plan included as part of medical record accessible by all members of care team and residents’ family members, including at discharge Accurate documentation of all nutrition interventions
Staff training and development	Targeted education and training of interdisciplinary staff on malnutrition risk, assessment, and diagnostic criteria as well as on opportunities for nutrition intervention

## Data Availability

Not applicable.

## Appendix A

**Table A1:** Skilled Nursing Facility (SNF) Patient Driven Payment Model (PDPM) case-mix adjusted components that include nutrition-related diagnoses and conditions.

Case-Mix Adjusted Component and Nutrition-Related Diagnoses/Conditions	Description
Speech Language Pathology (SLP)	
Swallowing disorder	Including loss of liquids/solids from mouth when eating/drinking, holding food in mouth/cheeks, coughing/choking during meals or when swallowing medications, complaints of difficulty/pain with swallowing
Mechanically-altered diet	Diet specifically prepared to alter texture/consistency to facilitate oral intake
**Non-therapy Ancillary (NTA)**	
Feeding tube	Includes nasal or abdominal tube
Malnutrition	Physician must document diagnosis of malnutrition
Morbid obesity	Body Mass Index (BMI) greater than or equal to 40 kg/m^2^
Parenteral intravenous (IV) feeding level high	51% or more of total calories received through IV feeding
Parenteral IV feeding level low	26–50% of total calories received through IV feeding and average fluid intake by IV or feeding tube of 501 cc per day or more
